# Prognostic model development for classification of colorectal adenocarcinoma by using machine learning model based on feature selection technique boruta

**DOI:** 10.1038/s41598-023-33327-4

**Published:** 2023-04-19

**Authors:** Neha Shree Maurya, Shikha Kushwah, Sandeep Kushwaha, Aakash Chawade, Ashutosh Mani

**Affiliations:** 1 Department of Biotechnology, Motilal Nehru National Institute of Technology Allahabad, Prayagraj, 211004 India; 2 National Institute of Animal Biotechnology, Hyderabad, 500032 India; 3 Department of Plant Breeding, Swedish University of Agricultural Sciences, 230 53 Alnarp, Sweden

**Keywords:** Cancer, Computational biology and bioinformatics, Gastroenterology, Oncology

## Abstract

Colorectal cancer (CRC) is the third most prevalent cancer type and accounts for nearly one million deaths worldwide. The CRC mRNA gene expression datasets from TCGA and GEO (GSE144259, GSE50760, and GSE87096) were analyzed to find the significant differentially expressed genes (DEGs). These significant genes were further processed for feature selection through boruta and the confirmed features of importance (genes) were subsequently used for ML-based prognostic classification model development. These genes were analyzed for survival and correlation analysis between final genes and infiltrated immunocytes. A total of 770 CRC samples were included having 78 normal and 692 tumor tissue samples. 170 significant DEGs were identified after DESeq2 analysis along with the topconfects R package. The 33 confirmed features of importance-based RF prognostic classification model have given accuracy, precision, recall, and f1-score of 100% with 0% standard deviation. The overall survival analysis had finalized GLP2R and VSTM2A genes that were significantly downregulated in tumor samples and had a strong correlation with immunocyte infiltration. The involvement of these genes in CRC prognosis was further confirmed on the basis of their biological function and literature analysis. The current findings indicate that GLP2R and VSTM2A may play a significant role in CRC progression and immune response suppression.

## Introduction

Colorectal cancer (CRC) occurs within the colon and rectum part of the digestive system^1^. According to the globocan 2020 cancer data, CRC is the third most common cancer and has an incidence rate of 10% with more than 1.93 million cases worldwide. In terms of mortality, it is the second most common cancer type accounting for approximately 0.93 million (9.4%) deaths worldwide.

Even though there have been advancements in the detection and treatment of CRC in the past decades, but still the 5-year survival rate of CRC is unsatisfactory^2^. Owing to the mentioned difficulties there is a strong need for the identification of new gene signatures which can differentiate between metastatic vs. non-metastatic CRC cells^3^.

The conventional prognostic models which were based on clinical predictors such as gender, age, and tumor-node-metastasis (TNM) staging were not precise in predicting CRC patients' survival due to its heterogeneous behaviour. Thus, for the establishment of novel predictive signatures, gene expression information can be of great importance^4^.

Studies have found that prognostic models which were developed by using gene expression count data had better accuracy in CRC prognosis and they helped in providing better and more effective therapy to high-risk patient groups^5,6^. Neogenin-1 (NEO1), which is a tumor suppressor gene was identified to be correlated with CRC progression. NEO1 mRNA gene expression was significantly reduced in CRC tumor tissues than in the adjacent tissues of clinical samples^7^. The overexpression of interleukin-6 (IL-6) is associated with the relapse of colon cancer^8^. Xiong et al. have suggested that overexpression of CXCL3 was associated with advanced tumor stage, distant metastasis, and lymphatic invasion^9^.

In a previous recent study, Ding et al.^10^ used the gene expression profiling method for the identification of core gene expression signatures for CRC. However, they included only microarray data of CRC with a single hub gene identification method. In this study, we used RNA-Seq data from Gene Expression Omnibus (GEO) and The Cancer Genome Atlas (TCGA) for the identification of significant genes based on their gene expression profile, and a feature selection technique was employed. Overall survival (OS) analysis and immunocyte infiltration analysis of the final set of genes was also performed on the TCGA (COAD and READ) cohorts.

## Results

### Gene expression data collection

**Figure 1:**
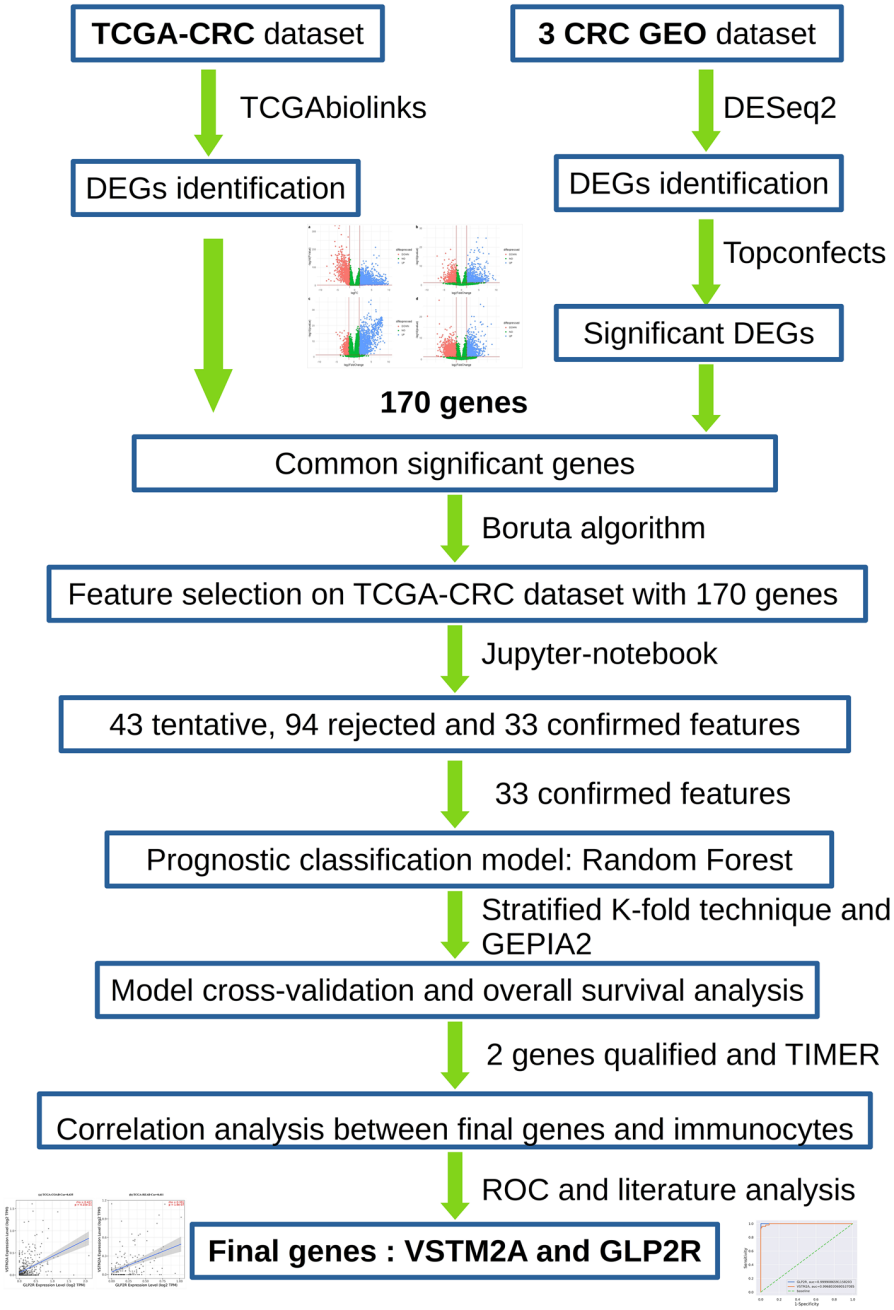
Workflow for identifying gene signatures for colorectal cancer. *TCGA* The Cancer Genome Atlas, *CRC* colorectal cancer, *GEO* gene expression omnibus, *DEGs* differentially expressed genes, *DAVID* database for annotation, visualization, and integrated discovery, *GEPIA* gene expression profiling interactive analysis.

The TCGA-CRC dataset was downloaded containing a total of 695 samples (644—tumor tissue and 51—normal tissue) from NIH-GDC. GEO datasets (a) GSE144259, (b) GSE50760, and (c) GSE87096 had a total of 75 samples that were included in the study. The overall sample distribution between different datasets is shown in Table 1. The workflow of our study is shown in Fig. 1.

**Table 1:** Sample distribution of TCGA-CRC and GEO datasets for normal tissue and tumor tissue samples.

Dataset	No. of normal tissue samples	No. of tumor tissue samples	Total samples
TCGA-CRC	51	644	695
GSE144259	3	6	9
GSE50760	18	36	54
GSE87096	6	6	12
All datasets	78	692	770

### Identification of significant DEGs

TCGA-CRC dataset had a total of 2933 DEGs which includes 1832 upregulated and 1101 downregulated genes. While the number of DEGs obtained after DESeq2 analysis for all GEO datasets is shown in Table 2.

**Table 2:** Total number of DEGs in all the CRC datasets.

Dataset	Upregulated	Downregulated	Total DEGs
TCGA-CRC	1832	1101	2933
GSE144259	675	486	1161
GSE50760	1410	969	2379
GSE87096	870	1492	2362

**Figure 2:**
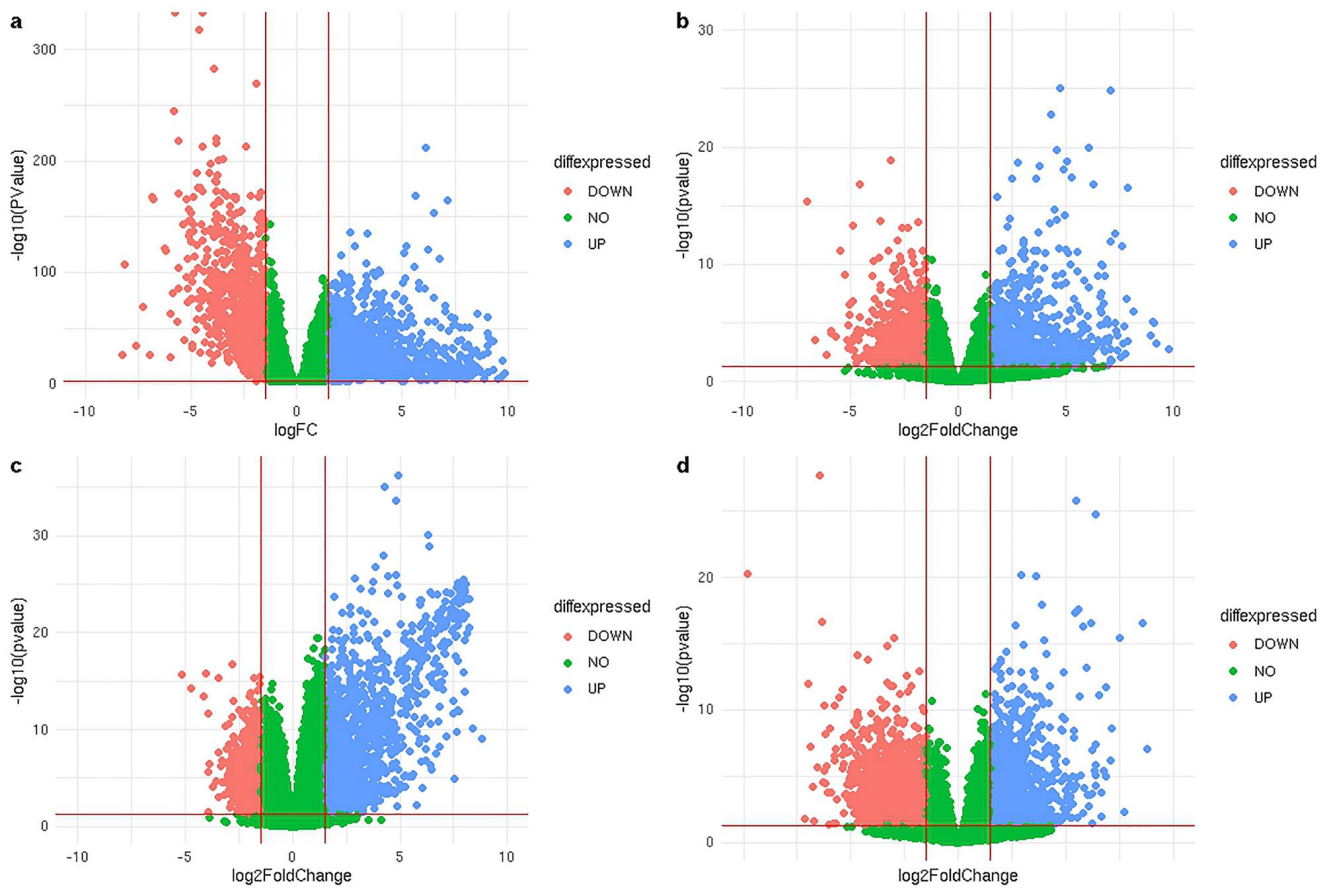
The volcano plot for distribution of DEGs in all CRC datasets. (**a**) TCGA-CRC, (**b**) GSE144259, (**c**) GSE50760, and (**d**) GSE87096. Green—non-significant DEGs, Red—upregulated genes, and Blue—downregulated genes.

**Figure 3:**
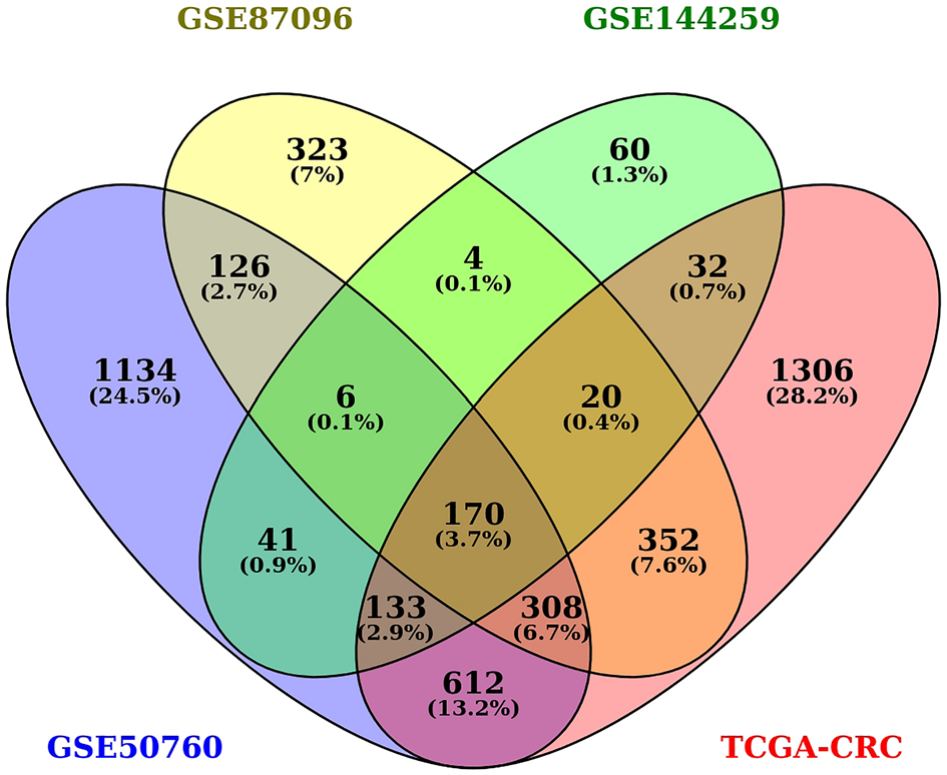
Venn diagram for a common significant number of genes between all four CRC datasets.

The distribution of all DEGs including significant and nonsignificant ones have been shown through volcano plot for all the datasets included in our study in Fig. 2. Topconfects had provided 466, 2530, and 1309 for GSE144259, GSE50760, and GSE87096, respectively with a step value of 0.5. A total of 170 significant common DEGs were found between 3 GEO datasets and the TCGA-CRC dataset as shown in Fig. 3.

### Feature selection and prognostic model development

The boruta provided 33 confirmed features for the classification of the TCGA-CRC data between normal and tumor class based on the gene expression data after 10 iterations. A total of 43 (ranked as 2) tentative and 94 rejected and 33 (ranked as 1) features were confirmed to be used for further analysis as shown in Table 3.

**Table 3:** List of rejected, tentative and confirmed features selected through boruta algorithm.

Rejected features (Ensembl Gene Id)	Gene names of rejected features	Tentative features (Ensembl Gene Id)	Gene names of tentative features	Confirmed features (Ensembl Gene Id)	Gene names of confirmed features
ENSG00000185479	KRT6B	ENSG00000137673	MMP7	ENSG00000163347	CLDN1
ENSG00000167755	KLK6	ENSG00000165905	LARGE2	ENSG00000122641	INHBA
ENSG00000105664	COMP	ENSG00000181652	ATG9B	ENSG00000060718	COL11A1
ENSG00000111700	SLCO1B3	ENSG00000101187	SLCO4A1	ENSG00000175832	ETV4
ENSG00000184937	WT1	ENSG00000185269	NOTUM	ENSG00000183034	OTOP2
ENSG00000170369	CST2	ENSG00000198759	EGFL6	ENSG00000105989	WNT2
ENSG00000159263	SIM2	ENSG00000164379	FOXQ1	ENSG00000164283	ESM1
ENSG00000160862	AZGP1	ENSG00000099953	MMP11	ENSG00000062038	CDH3
ENSG00000115363	EVA1A	ENSG00000170373	CST1	ENSG00000167767	KRT80
ENSG00000105219	CNTD2	ENSG00000138028	CGREF1	ENSG00000181577	C6orf223
ENSG00000108244	KRT23	ENSG00000103257	SLC7A5	ENSG00000142959	BEST4
ENSG00000134762	DSC3	ENSG00000173898	SPTBN2	ENSG00000168748	CA7
ENSG00000196611	MMP1	ENSG00000103888	CEMIP	ENSG00000178773	CPNE7
ENSG00000184292	TACSTD2	ENSG00000169429	CXCL8	ENSG00000101255	TRIB3
ENSG00000113739	STC2	ENSG00000044012	GUCA2B	ENSG00000129474	AJUBA
ENSG00000172031	EPHX4	ENSG00000157193	LRP8	ENSG00000112559	MDFI
ENSG00000135480	KRT7	ENSG00000164932	CTHRC1	ENSG00000101115	SALL4
ENSG00000165376	CLDN2	ENSG00000173894	CBX2	ENSG00000131389	SLC6A6
ENSG00000139515	PDX1	ENSG00000186007	LEMD1	ENSG00000120254	MTHFD1L
ENSG00000107807	TLX1	ENSG00000163295	ALPI	ENSG00000187783	TMEM72
ENSG00000158296	SLC13A3	ENSG00000183742	MACC1	ENSG00000065325	GLP2R
ENSG00000133048	CHI3L1	ENSG00000005001	PRSS22	ENSG00000162706	CADM3
ENSG00000129451	KLK10	ENSG00000186198	SLC51B	ENSG00000135549	PKIB
ENSG00000137699	TRIM29	ENSG00000071539	TRIP13	ENSG00000104267	CA2
ENSG00000175592	FOSL1	ENSG00000197905	TEAD4	ENSG00000197273	GUCA2A
ENSG00000165816	VWA2	ENSG00000253958	CLDN23	ENSG00000165072	MAMDC2
ENSG00000088992	TESC	ENSG00000122778	KIAA1549	ENSG00000166869	CHP2
ENSG00000009950	MLXIPL	ENSG00000111110	PPM1H	ENSG00000133742	CA1
ENSG00000166415	WDR72	ENSG00000006704	GTF2IRD1	ENSG00000163815	CLEC3B
ENSG00000183734	ASCL2	ENSG00000170382	LRRN2	ENSG00000170419	VSTM2A
ENSG00000273079	GRIN2B	ENSG00000036672	USP2	ENSG00000079689	SCGN
ENSG00000135069	PSAT1	ENSG00000105976	MET	ENSG00000152785	BMP3
ENSG00000139289	PHLDA1	ENSG00000187699	C2orf88	ENSG00000196950	SLC39A10
ENSG00000081041	CXCL2	ENSG00000160191	PDE9A		
ENSG00000169248	CXCL11	ENSG00000114346	ECT2		
ENSG00000169247	SH3TC2	ENSG00000172594	SMPDL3A		
ENSG00000131746	TNS4	ENSG00000137872	SEMA6D		
ENSG00000103375	AQP8	ENSG00000088836	SLC4A11		
ENSG00000115507	OTX1	ENSG00000124205	EDN3		
ENSG00000128683	GAD1	ENSG00000154175	ABI3BP		
ENSG00000015413	DPEP1	ENSG00000168309	FAM107A		
ENSG00000172927	MYEOV	ENSG00000162817	C1orf115		
ENSG00000155850	SLC26A2	ENSG00000122694	GLIPR2		
ENSG00000099194	SCD				
ENSG00000171004	HS6ST2				
ENSG00000119121	TRPM6				
ENSG00000008300	CELSR3				
ENSG00000168060	NAALADL1				
ENSG00000173175	ADCY5				
ENSG00000165188	RNF183				
ENSG00000068650	ATP11A				
ENSG00000101057	MYBL2				
ENSG00000120875	DUSP4				
ENSG00000136997	MYC				
ENSG00000078114	NEBL				
ENSG00000163191	S100A11				
ENSG00000197766	CFD				
ENSG00000197165	SULT1A2				
ENSG00000173557	C2orf70				
ENSG00000197275	RAD54B				
ENSG00000157005	SST				
ENSG00000118777	ABCG2				
ENSG00000088002	SULT2B1				
ENSG00000117394	SLC2A1				
ENSG00000137203	TFAP2A				
ENSG00000141682	PMAIP1				
ENSG00000109084	TMEM97				
ENSG00000112877	CEP72				
ENSG00000204335	SP5				
ENSG00000117122	MFAP2				
ENSG00000101144	BMP7				
ENSG00000259823	LYPD8				
ENSG00000174371	EXO1				
ENSG00000196196	HRCT1				
ENSG00000168447	SCNN1B				
ENSG00000163734	CXCL3				
ENSG00000215182	MUC5AC				
ENSG00000174358	SLC6A19				
ENSG00000151012	SLC7A11				
ENSG00000181544	FANCB				
ENSG00000141574	SECTM1				
ENSG00000163739	CXCL1				
ENSG00000101850	GPR143				
ENSG00000170312	CDK1				
ENSG00000082397	EPB41L3				
ENSG00000078804	TP53INP2				
ENSG00000164176	EDIL3				
ENSG00000135916	ITM2C				
ENSG00000164109	MAD2L1				
ENSG00000183960	KCNH8				
ENSG00000176641	RNF152				
ENSG00000168016	TRANK1				
ENSG00000164442	CITED2				

**Figure 4:**
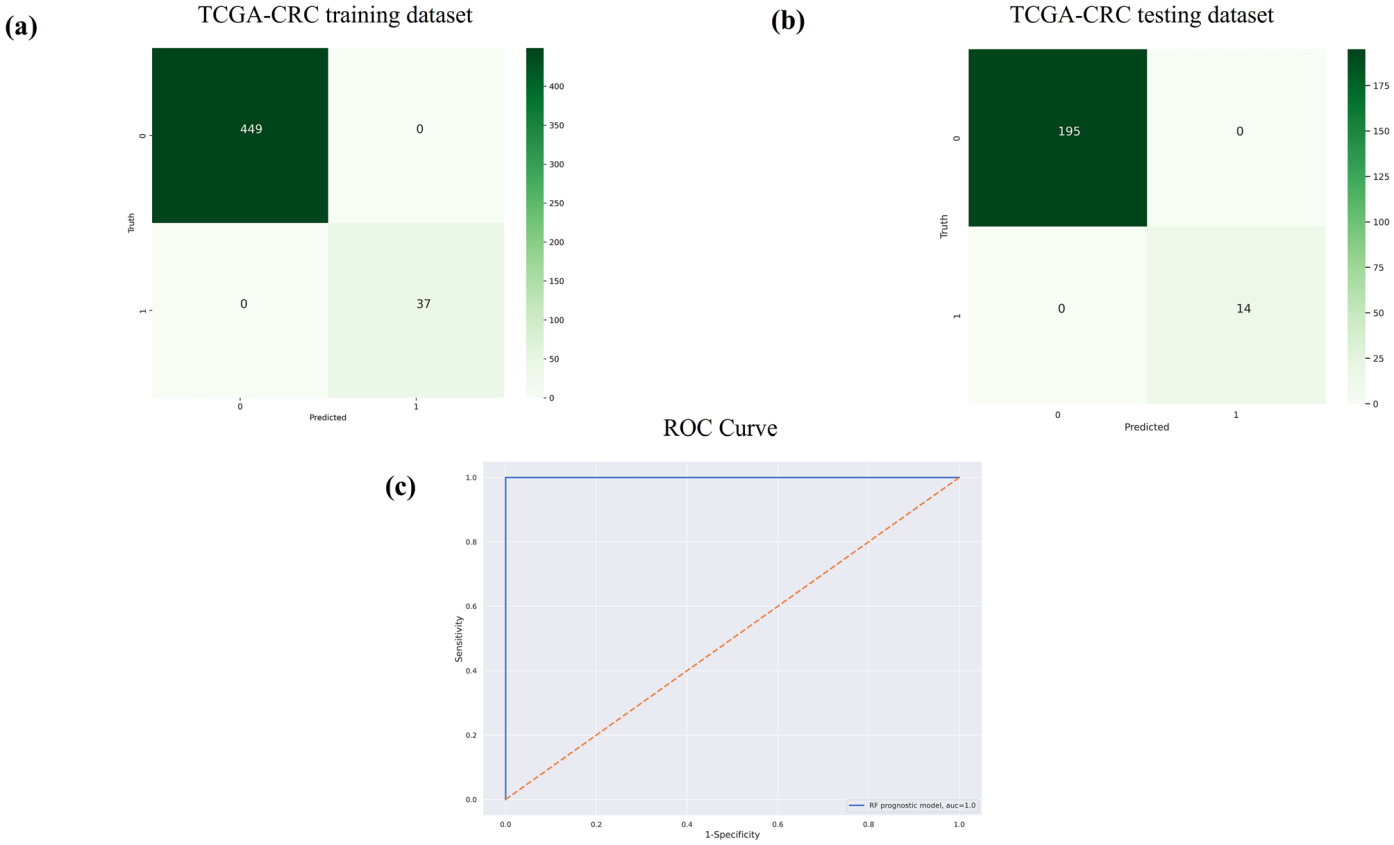
Random Forest based prognostic model performance analysis. (**a**) Confusion matrix of the training dataset between the truth and predicted values with 486 samples of TCGA-CRC dataset, (**b**) confusion matrix of the testing dataset between the truth and predicted values with 209 samples of TCGA-CRC dataset, and (**c**) ROC curve for RF based prognostic model with 33 features identified through boruta feature selection algorithm. 0 denotes tumor class while 1 denotes normal class sample numbers.

The Random Forest (RF) classifier was implemented on the gene expression data of these 33 confirmed features. An accuracy score of 100% was obtained for this RF-based prognostic model. The performance metrics for the RF-based prognostic model is provided in Table 4 which shows the 100% score for both the sensitivity and positive predictive value. The confusion matrix of the training and testing dataset is provided in Fig. 4a,b, respectively and the ROC analysis for the model with an AUC curve is shown in Fig. 4c which shows the predicted and truth class labels with their classification values for tumor and normal sample classes.

**Table 4:** Classification performance report of TCGA-CRC dataset on the training and testing dataset with 486 and 209 samples, respectively for the prognostic random forest based ML model.

Sample class	Number of CRC samples	Precision (PPV)	Recall (sensitivity)	f1-score
Training dataset				
0 (Tumor)	449	100%	100%	100%
1 (Normal)	37	100%	100%	100%
Testing dataset				
0 (Tumor)	195	100%	100%	100%
1 (Normal)	14	100%	100%	100%

*PPV* positive predictive value.

### Prognostic model cross-validation and survival analysis

**Figure 5:**
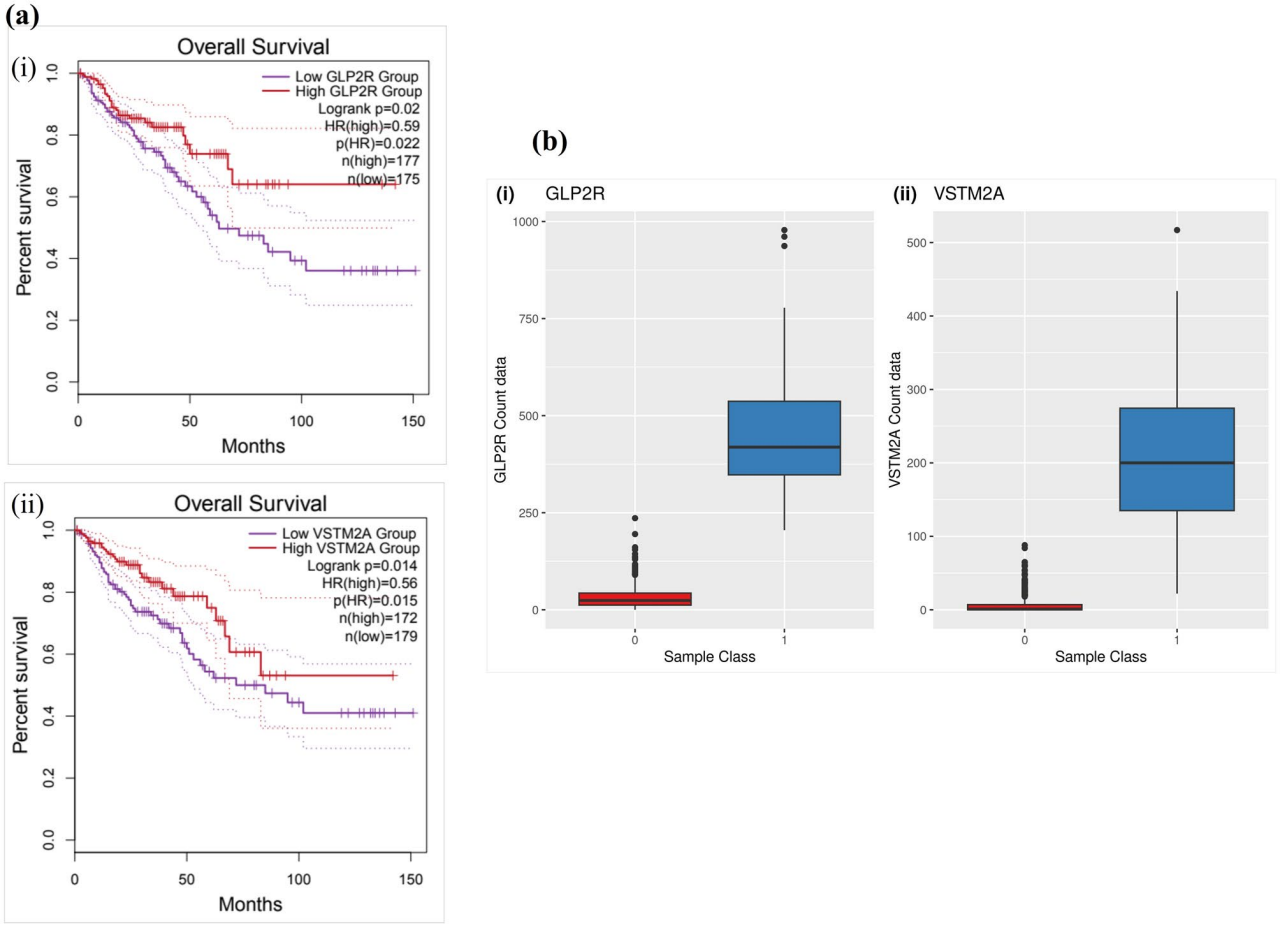
Overall survival analysis and gene expression profiling of CRC samples. (**a**) Kaplan–Meier plot for overall survival analysis of GLP2R and VSTM2A genes between low risk and high-risk groups having log-rank value p < 0.05, and (**b**) Gene expression analysis for GLP2R and VSTM2A genes from the TCGA-CRC dataset. The blue color denotes the normal tissue samples while the red color denotes the tumor tissue samples.

The stratified K-Fold method has provided the list of possible accuracy, maximum accuracy, minimum accuracy, and standard deviation of the developed prognostic model for 33 confirmed features. The model has achieved 100% accuracy (maximum and minimum) with 0% standard deviation. The overall survival analysis was performed for the 33 confirmed features and only 2 genes namely GLP2R, and V-set and transmembrane domain containing 2A (VSTM2A) were identified which had log-rank p < 0.05. The GLP2R had log-rank p = 0.02 and VSTM2A had log-rank p = 0.014, respectively as shown in Fig. 5a. The gene expression analysis based on count data information shows significant downregulation of tumor class for GLP2R and VTSM2A genes when compared with normal class analyzing TCGA-CRC dataset as shown in Fig. 5b.

**Figure 6:**
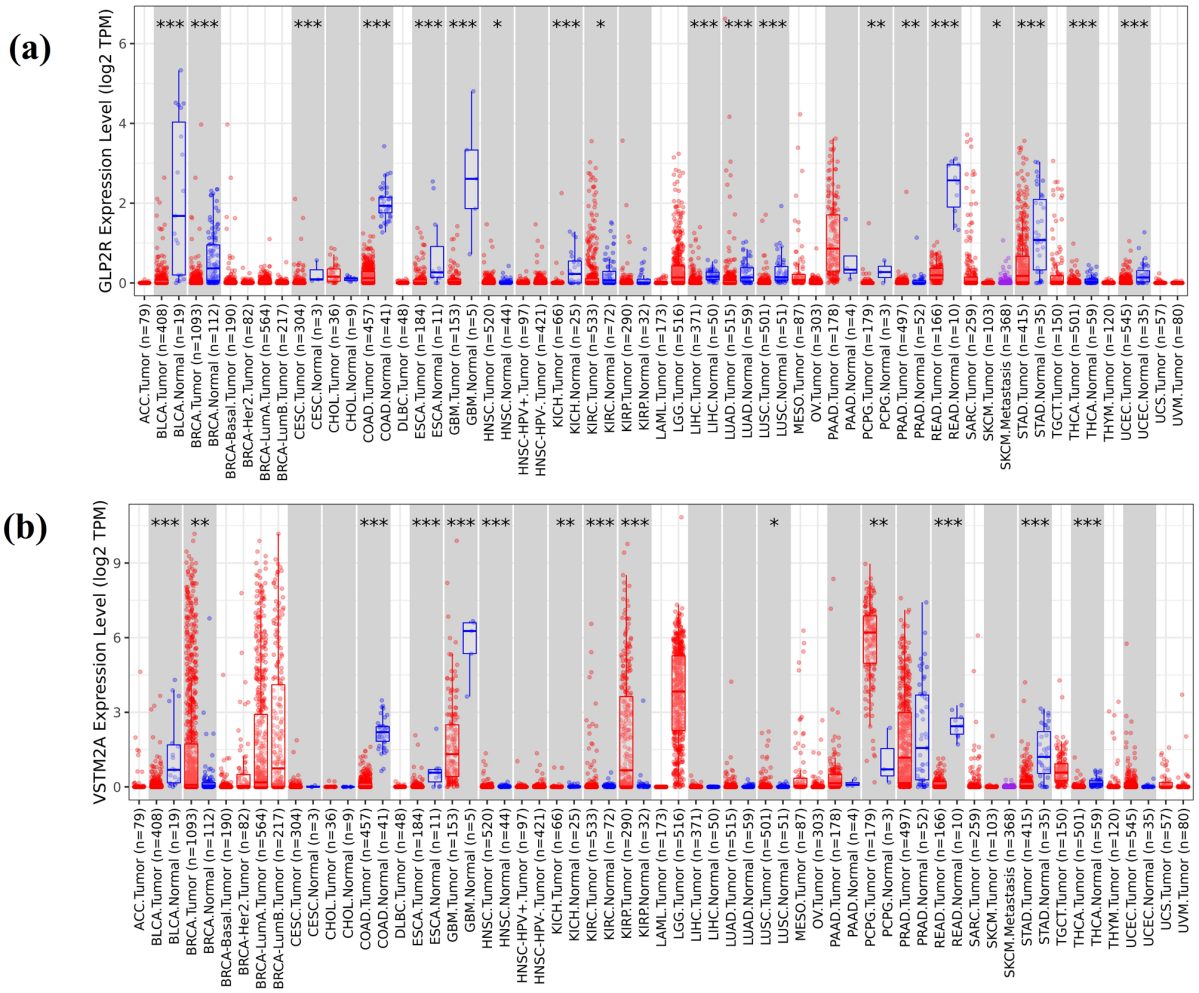
The expression of final set of genes (GLP2R and VSTM2A) in tumor and normal tissue, where red box plots and blue box plots suggests tumor tissues and normal tissues across different cancer types respectively. “***” indicated P < 0.001.

The gene expression profile of GLP2R and VSTMA genes across TCGA repository for different cancer types is shown in Fig. 6. It is visible that the expression of GLP2R and VSTM2A is downregulated in CRC datasets while the expression in normal samples is upregulated.

### Correlation analysis between the final set of genes and immunocytes

**Figure 7:**
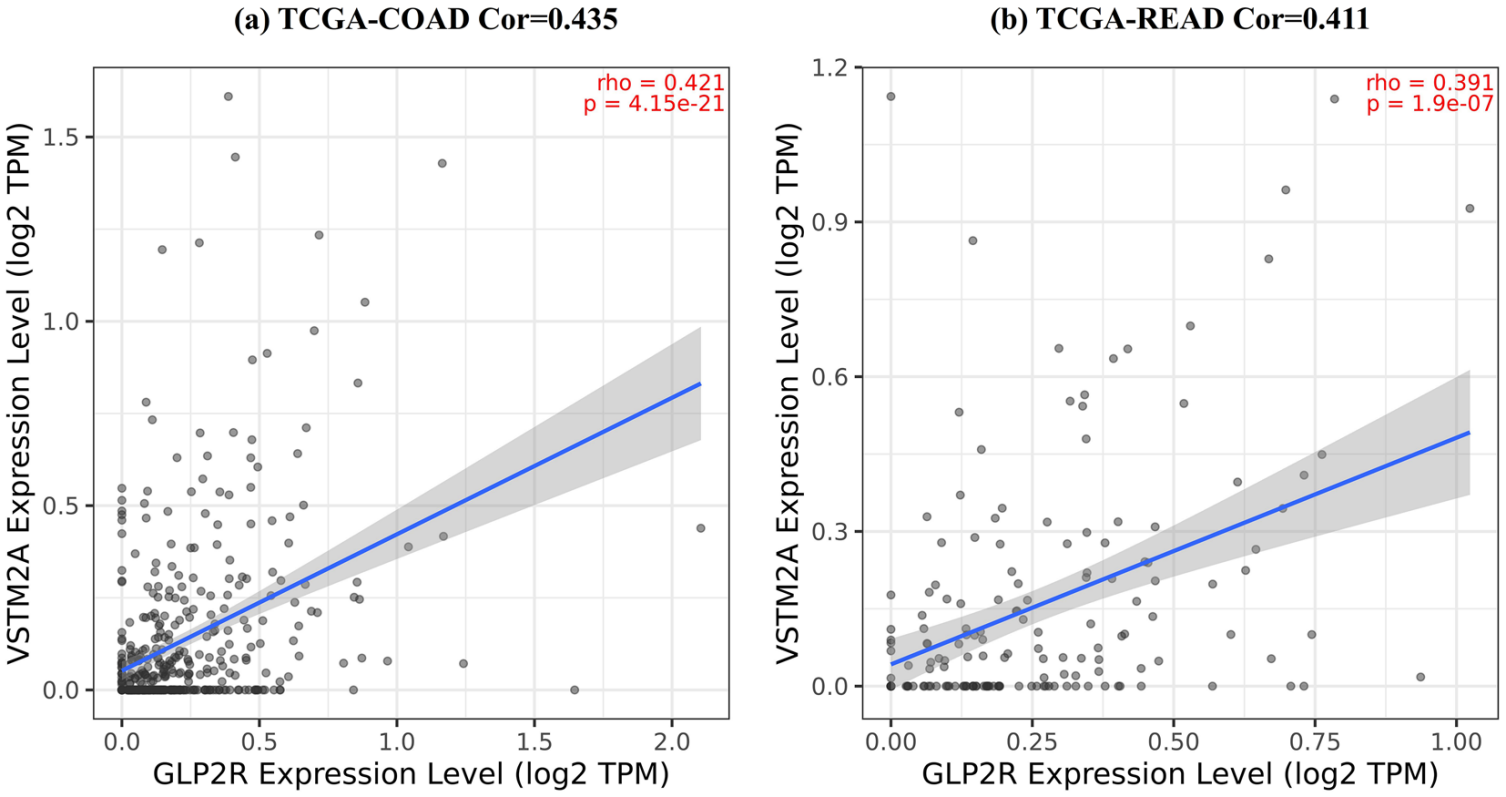
Correlation analysis of GLP2R and VSTM2A for TCGA-COAD and TCGA-READ samples. It shows strong positive and statistically significant correlation between both TCGA datasets. (**a**) Correlation value of 0.435 for TCGA-COAD samples, and (**b**) Correlation value of 0.411 for TCGA-READ samples.

The correlation analysis between the final set of genes GLP2R and VSTM2A has shown a positive correlation for TCGA-CRC datasets through TIMER. The correlation value for COAD and READ datasets are 0.435 and 0.411, respectively as shown in Fig. 7.

**Figure 8:**
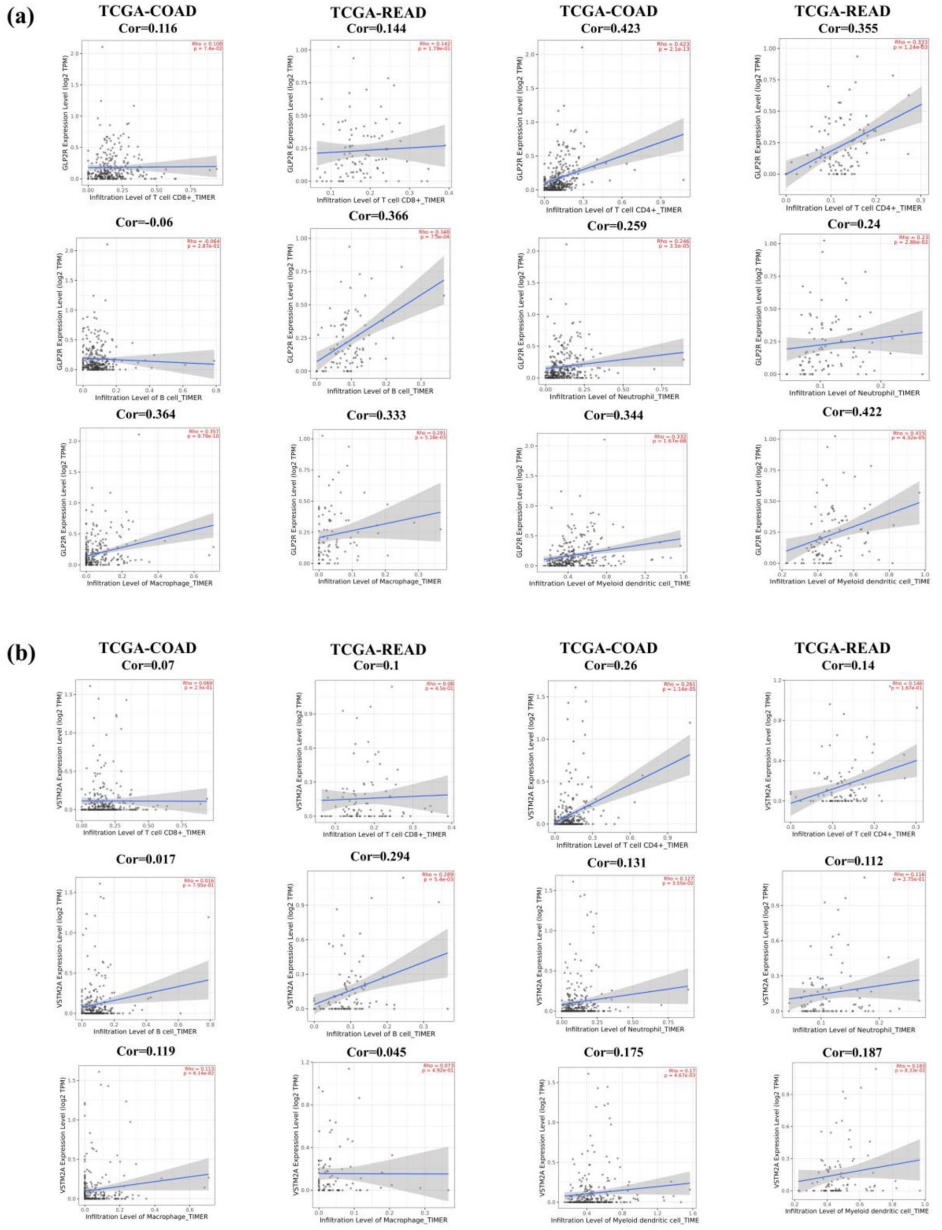
Correlation of final set of genes with immunocyte infiltration. (**a**) The correlation plot of GLP2R and (**b**) VSTM2A with immunocytes for TCGA-COAD and TCGA-READ datasets.

The correlation analysis for the GLP2R and VSTM2A genes with immunocytes was also analyzed and was found that there is a strong correlation between TIIC with CRC as shown in Fig. 8a,b. The maximum correlation for GLP2R (TCGA-COAD cor = 0.423, TCGA-READ cor = 0.355) and VSTM2A (TCGA-COAD cor = 0.26, TCGA-READ cor = 0.14) gene expression with immunocytes infiltration level was found with CD4 + T-cells and the minimum was found GLP2R (TCGA-COAD cor = 0.116, TCGA-READ cor = 0.144) and VSTM2A (TCGA-COAD cor = 0.07, TCGA-READ cor = 0.1) for CD8 + T-cells.

### ROC curve and literature analysis

**Figure 9:**
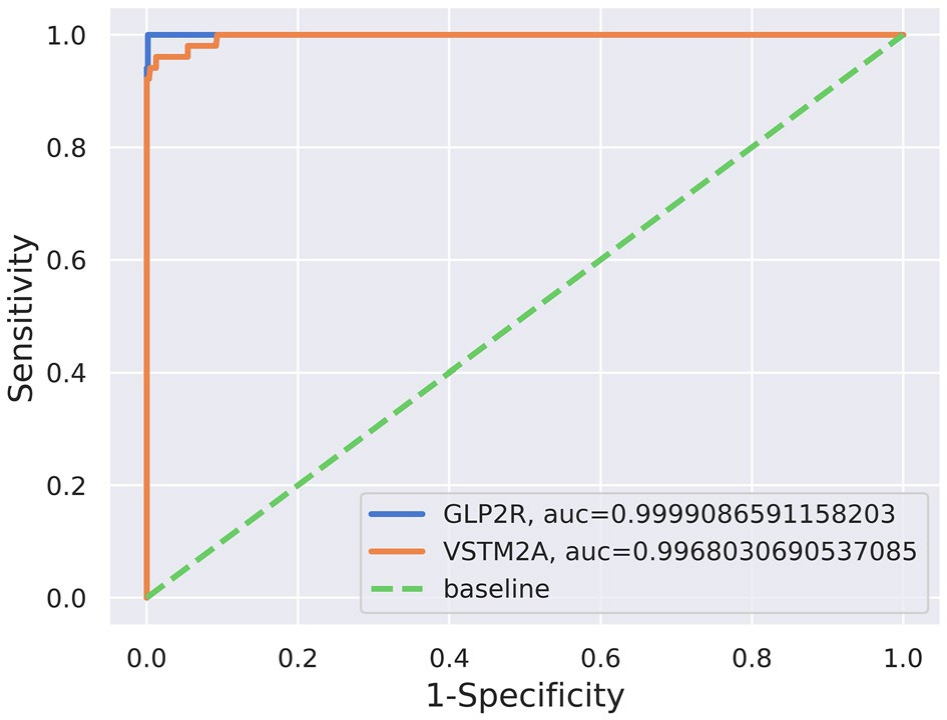
ROC curve for the finalized features GLP2R and VSTM2A in classifying the tumor and normal classes of CRC dataset. The blue color denotes the AUC for GLP2R gene, red color denotes the VSTM2A gene, and the green color is baseline for the sample classification.

The significant features GLP2R and VSTM2A have shown the ROC curve analysis with an area under the curve (AUC) of 99.99% and 99.68%, respectively with RF-based prognostic model as shown in Fig. 9. Since the GLP2R and VSTM2A had shown significant downregulation in tumor cells their biological functions which would get affected are mentioned in Table 5.

**Table 5:** Literature analysis for biological function of GLP2R and VSTM2A genes.

Final features (genes)	Biological functions
GLP2R (glucagon like peptide 2 receptor)	Encodes a G protein-coupled receptor that is closely related to the glucagon receptor and binds to glucagon-like peptide-2 (GLP2). Signalling through GLP2 stimulates intestinal growth and increases villus height in the small intestine, concomitant with increased crypt cell proliferation and decreased enterocyte apoptosis
VSTM2A (V-set and transmembrane domain containing 2A)	regulation of the early stage of white and brown preadipocyte cell differentiation

The brainstem, hypothalamus, stomach, colon, ileum, jejunum, lung, and duodenum widely express GLP2R and its ligand GLP2 helps in stimulating the GLP2R activity in the intestinal subepithelial fibroblasts (ISEMFs). The release of growth factors after GLP2R activation promotes colonic epithelial proliferation^24^. The downregulation of VSTM2A is linked with poor survival of CRC patients because it acts as a novel antagonist of the Wnt signaling pathway. The direct binding of VSTM2A with LDL receptor related protein 6 (LRP6) induces lysosome-mediated degradation and endocytosis of LRP6^25^.

## Discussion

Colorectal cancer is one of the leading causes of morbidity worldwide. The early diagnosis of CRC can be helpful in improving the survival rate of patients rather than diagnosing at a later stage^26^. Therefore, studies for identification of diagnostic and prognostic biomarkers with their predictive efficacy can be of great significance^27–29^.

A study conducted by sun et.al. identified a set of immune-related genes (IRGs) which were used to develop the prognostic model for the classification of colon adenocarcinoma^30^. The CTHRC1 was identified as a prognostic predictor for CRC which is a peritoneal metastasis-related gene^31^.

The current study used TCGA-CRC and 3 GEO CRC datasets for the identification of significant DEGs which will further help in the identification of immune-related gene signatures for CRC prognosis. A list of 170 significant common DEGs were identified through topconfects analysis along with the DESeq2 R package.

The 33 features of importance were obtained after the implementation of the feature selection technique boruta algorithm which is based on the working principle of RF although it can be used with other ML algorithms also. These 33 confirmed features of importance were utilized to develop a prognostic classification model through RF algorithm on the TCGA-CRC dataset for classifying the tumor and normal data. These 33 features gene expression count data for 695 CRC samples were used to divide for training and testing dataset in a 7:3 ratio. The gene expression dataset of CRC with 33 confirmed features have scored 100% accuracy with the test dataset containing 209 samples. The accuracy obtained through RF prognostic classification model was cross-validated through the stratified k-fold technique and the observed standard deviation was 0 which shows the robustness of the prognostic ML model. The overall survival analysis has provided 2 genes (out of 33 genes) GLP2R (log-rank p-value = 0.02) and VSTM2A (log-rank p-value = 0.014) which had passed the threshold of log-rank p-value < 0.05. The gene expression analysis has shown significant downregulation of both the genes and both GLP2R and VSTM2A genes have shown a positive correlation average value of 0.42 (TCGA-COAD and TCGA-READ). The ROC curve analysis with the area under the curve (AUC) for GLP2R and VSTM2A gene has shown the value of 99.99% and 99.68%, respectively.

Immunocyte infiltration analysis for GLP2R and VSTM2A has shown a positive correlation with TIIC. GLP2R is predominantly found in the gastrointestinal tract and is important for maintaining the integrity of the colonic epithelial cells. Studies have shown that GLP2R expression stimulates colonic epithelial cell proliferation and inhibits apoptotic cell death in the crypt compartment^32–35^. Gene expression analysis through TCGA-CRC data, GEPIA, and TIMER has shown the downregulation of GLP2R in the tumor samples as compared to the normal samples.

There are some known possible molecular mechanisms that promotes CRC tumorigenesis by binding GLP-2 to GLP2R. One of the mechanisms involves GLP-2 induced colon tumor promotion by stimulating insulin-like growth factor 1 (IGF-1) synthesis in intestinal subepithelial fibroblasts and its (GLP-2) own synthesis^36–38^. The GLP2 (secreted through colon tumor cells) binds to GLP2R expressed by carcinoma-related fibroblasts. CAFs are the cell phenotypes that composes the tumor microenvironment and play a significant role in the communication between the compartments of the epithelium and stroma. This leads to angiogenic enhancement, cytokine and growth factor secretion, and immune response suppression which enables tumor cells to expand, differentiate and invade^39,40^.

Another mechanism that stimulates CRC proliferation involves the phosphatidylinositol 3 kinase/protein kinase B (PI3-K/Akt) activation through Fibroblast-expressed IGF-1. The expression of IGF-1 is stimulated through Akt and subsequently, IGF-1 is released from the fibroblasts^41,42^. Following this, IGF-1 binds to colonocyte receptors and transmits signals via the PI3-K/Akt/GSK-3β pathway^38,43^. A study conducted on HT29 colon cancer cells has shown that IGF-1 stimulates the PI3-K activity which further induces Akt phosphorylation^44^. To increase the proliferation of tumor cells, GLP2 might boost the production of IGF-1, which, upon binding with IGF-1r in colon tumor cells, would activate Akt signalling.

VSTM2A regulates preadipocyte cell differentiation. The gene expression analysis has shown a significant reduction in tumor samples as compared to the normal CRC samples. According to studies, VSTM2A downregulation and VSTM2A DNA promoter hypermethylation are linked to poor prognosis of CRC patients and colorectal tumorigenesis is affected by the hyperactivation of the Wnt/β-catenin signalling pathway. The interaction between VSTM2A and LRP6 inhibits the Wnt signalling intracellularly. It occurs through suppressing LRP6 protein expression and inhibiting LRP6 phosphorylation, both of which are dose-dependently increased by the presence of VSTM2A protein^45^. According to studies, the binding of ligands to their receptor often induces receptor endocytosis^46^. Studies have shown that the interaction between VSTM2A and LRP6 induces the lysosome-mediated degradation and endocytosis of VSTM2A.

This study has certain limitations as it has been performed on the publicly available dataset and external real-time dataset validation will be required for the developed prognostic classification model.

## Conclusion

In recent years several studies have been designed for the early diagnosis of CRC with the assistance of technological advancements. This study utilizes the significant DEGs to further develop the ML-based prognostic classification model. The developed ML model has shown consistent performance with the cross-validation algorithm and has a 0% standard deviation. The finalized immune-related genes GLP2R and VSTM2A had shown a positive correlation with the immunocyte infiltration and have a role in the suppression of immune response. The biological, functional, and gene expression analysis has also proved the role of these genes in CRC progression.

## Methods

### CRC gene expression dataset collection

All CRC RNA-Seq datasets were downloaded from the TCGA and GEO databases. The TCGA-CRC gene expression dataset was downloaded from Genomic Data Commons Data Portal (NIH-GDC) https://portal.gdc.cancer.gov-/ through the R Bioconductor package TCGAbiolinks^11^. The TCGA-CRC mRNA gene expression contained 51 normal tissue samples and 644 tumor tissue samples (695 samples). The other RNA-Seq datasets were downloaded from GEO (https://www.ncbi.nlm.nih.gov/geo/). The GEO datasets which were incorporated in this study are (a) GSE144259^12^, (b) GSE50760^13–15^, and (c) GSE87096^16^.

### Identification of significant DEGs

TCGAbiolinks package was used to pre-process the TCGA-CRC mRNA gene expression data. The TCGA-CRC dataset genes which had correlation cutoff value less than 0.6 were removed for the DEG analysis by utilizing the command “dataPrep <-TCGAanalyze_Preprocessing(object = dataPrep, cor.cut = 0.6, datatype = "HTSeq – Counts")” and the normalization was performed through dataNorm <-TCGAanalyze_Normalization(tabDF = dataPrep, geneInfo = geneInfoHT, method = "gcContent"). Thereafter, the threshold implemented to identify the DEGs between normal and CRC tumor samples using the edgeR by glmRT method was FDR cut-off of 0.01 and |log 2‐FC|> 1.5.

The gene expression count data for GEO datasets were processed through DESeq2^17^ R Bioconductor package. The initial pre-filtering of the low count reads were performed through the rowsums count parameter function of the DESeq2 package by using the command : dds <-rowSums(counts(dds)) >  = 100. It filters the genes which had row sum value less than 100 in terms of gene counts. The GEO datasets normalization was performed through counts function by applying the R command: normalized_counts <-counts(dds, normalized = TRUE). Further, the DEGs for GEO datasets were identified by applying a threshold of adj. p-value < 0.05 and |log 2‐FC|> 1.5.

To obtain a significant list of DEGs from GEO datasets "topconfects"^18^ Bioconductor R package was employed which ranks the genes based on the confidence score bound to log fold change value. The deseq2_confects function was used on all GEO datasets with a step value of 0.5. The common shared significant genes list between TCGA-CRC and all GEO datasets after topconfects implementation were used for further analysis. The common genes list was analyzed by the venny tool^19^.

### Feature selection and prognostic model development

The gene expression data of 170 common features (genes) was used for developing a prognostic model by utilizing TCGA-CRC dataset. The jupyter-notebook platform was used for model implementation. The features of importance were identified through boruta feature selection^20^ algorithm, which was implemented through python library “BorutaPy” based on Random Forest^21^ which selects features by generating shadow attributes for the original set of features. The parameters which were used by boruta were number of iterations = 10 and number of estimators = 'auto'. The confirmed identified features were further used for developing a prognostic classification model by using RF algorithm utilizing the python library “RandomForestClassifier”. The training and testing dataset of TCGA-CRC was divided into a ratio of 7:3, respectively. To evaluate the performance of the prognostic model accuracy score, precision, recall, and f1-score were calculated by using confusion matrix information by utilizing “sklearn.metrics” python library.

### Prognostic model cross-validation and survival analysis

The prognostic ML classification model was cross-validated through the stratified K-Fold method utilizing the “StratifiedKFold” python library (number of splits = 4) to observe the performance consistency in terms of accuracy and statistical significance for TCGA-CRC dataset classification. The confirmed features were further analyzed for survival analysis through GEPIA2 online server (http://gepia2.cancer-pku.cn/)^22^ based on the threshold of log-rank p value < 0.05. The features that had passed the before-mentioned threshold were analyzed for their gene expression count data.

### Correlation analysis between the final set of genes and immunocytes

Tumor Immune Estimation Resource (TIMER) is a publicly available web resource for analyzing tumor-infiltrating immune cells (TIIC) across different cancer types. It includes 10,897 samples for 32 cancer types from the TCGA repository. It is available at https://cistrome.shinyapps.io/timer/ and TIIC includes an abundance of CD4 + T-cells, CD8 + T-cells, B cells, neutrophils, macrophages, and dendritic cells. In this study, the correlation analysis of TIIC abundance concerning 2 finalized genes in TCGA-COAD and TCGA-READ was explored^23^.

### ROC Curve and literature analysis

The identified significant features for CRC classification were further analyzed for their receiver-operating characteristic (ROC) curve analysis by using the TCGA-CRC dataset gene expression count with the help of “skelarn.metrics” python library. The biological functional analysis of the final set of features was also performed through a literature survey.

## Data Availability

Publicly available dataset was used analyzed which are available on the TCGA repository site (https://portal.gdc.cancer.gov/) with project name TCGA-COAD and TCGA-READ and GEO site (https://www.ncbi.nlm.nih.gov/geo/). The accession number for the GEO datasets are (a) GSE144259, (b) GSE50760, and (c) GSE87096.
